# Effects of menstruation on the onset of acute coronary syndrome in premenopausal women: A case series

**DOI:** 10.1016/j.crwh.2023.e00486

**Published:** 2023-02-13

**Authors:** Marte F. van der Bijl, Madoka Sunamura, Nienke ter Hoeve, Michelle M. Schreuder, Mattie J. Lenzen, Jeanine E. Roeters van Lennep

**Affiliations:** a Capri Cardiac Rehabilitation, Rotterdam, the Netherlands; b Department of Internal Medicine, Erasmus MC University Medical Center, Rotterdam, the Netherlands; c Department of Cardiology, Thoraxcenter, Erasmus University Medical Center, Rotterdam, the Netherlands

**Keywords:** Menstrual cycle, Acute coronary syndrome, Premenopausal women

## Abstract

**Background:**

The incidence of cardiovascular disease (CVD) among women is lower before the menopause, which may be due to the atheroprotective effects of female sex hormones, including estrogens. This study explored whether women experienced acute coronary syndrome (ACS) more often during menstruation, when the levels of female sex hormones are low.

**Methods:**

All premenopausal women referred to the local cardiac rehabilitation program after ACS between August 2010 and September 2018 were contacted by telephone to gather information about their menstrual cycle, contraceptive use and whether ACS occurred during menstruation. Information on cardiovascular risk factors was collected using the clinical electronic health record.

**Results:**

Of the 22 women fulfilling the inclusion criteria and having a regular menstrual cycle, 22.7% reported that they were diagnosed with ACS at the time of menstruation.

**Conclusions:**

The percentage of women who were menstruating whilst having their cardiovascular event is higher than the percentage expected if the event was unrelated to the menstrual cycle. To gain more insight into the effect of female sex hormones on ACS, it is suggested that information on the menstrual cycle is routinely collected from women admitted to hospital with the condition.

## Highlights

• Female sex hormones may have a protective role against acute coronary syndrome.
• Women may have a higher risk of experiencing acute coronary syndrome during menstruation.
• Routine enquiry about the menstrual cycle at the time of hospital admission is needed.

## 1 Introduction

Cardiovascular disease (CVD) is the leading cause of mortality for women worldwide, accounting for 35% of total deaths in women in 2019 [1]. Although cardiovascular mortality for both men and women has decreased over the last decades, reductions in mortality from coronary heart disease (CHD) have been larger in men than in women [2]. Since there is more awareness of the sex differences in CHD, there has been a decline in mortality in women, but still there seems to be a gap in knowledge of the sex-specific coronary pathophysiology underlying CHD [3].

In general, women are older when they experience their first cardiovascular event [4]. Although fewer women than men get acute coronary syndrome (ACS) at a young age (< 55 years), the negative impact of ACS on quality of life in this group of young women is significantly higher than in young men [5]. The low incidence of CHD among women of reproductive age is likely due to the protective role of female sex hormones such as estrogen and progesterone [6,7]. During menstruation (days 1–4 of the menstrual cycle) the levels of estrogen are at their lowest [8]. Therefore, women may have a higher risk of experiencing ACS in this period.

The aim of this case series is to explore whether premenopausal women are at greater risk of developing ACS during menstruation compared with the rest of the menstrual cycle.

## 2 Methods

### 2.1 Study Sample

Between August 2010 and September 2018 all consecutive patients referred to Capri CR Center for a standard cardiac rehabilitation program after confirmed ACS and fulfilling the following criteria were eligible for inclusion in this research: a confirmed diagnosis of ACS [9], age < 55 years and proficiency in Dutch or English. Exclusion criteria were irregular or absent menstrual periods, hormonal contraceptive use, and inability to recall the phase of their menstrual cycle at the time of ACS.

The research was conducted in accordance with the Declaration of Helsinki [10]. The Erasmus MC Medical Ethics Committee decided that according to the Dutch Human Research Law (WMO), the protocol required no formal ethical approval. All patients provided written informed consent.

### 2.2 Definition of Acute Coronary Syndrome

ACS was defined as persistent (>20 min) chest pain suggestive of myocardial ischemia, which is unresponsive to nitroglycerine and is accompanied by ST-T changes (electrocardiographic evidence) or cardiac troponin elevations (biochemical evidence), regardless of in-hospital treatment [9].

### 2.3 Definition of Menstrual Cycle and Menstruation

Menstruation is defined as days 1–4 of a 28-day menstrual cycle [11].

### 2.4 Data Collection

Patients who participated in the standard cardiac rehabilitation program at Capri CR Center and fulfilled the inclusion criteria were invited for a telephone interview. The interview was based on a questionnaire (see Appendix A) focusing on regularity of menstrual cycle at the time of the event (if not, for what reason), the usage of contraception, and whether they were menstruating at the time of ACS. Information on cardiovascular risk factors (a history of CVD, diabetes, smoking, obesity, hypertension, and a family history of CVD) was collected using the electronic health record of the Capri CR Center.

### 2.5 Statistical Analysis

Since this concerns an exploratory study with a small sample size, only descriptive analyses were carried out, using IBM SPSS Statistics for Macintosh, Version 27.0.

## 3 Results

**Fig. 1:**
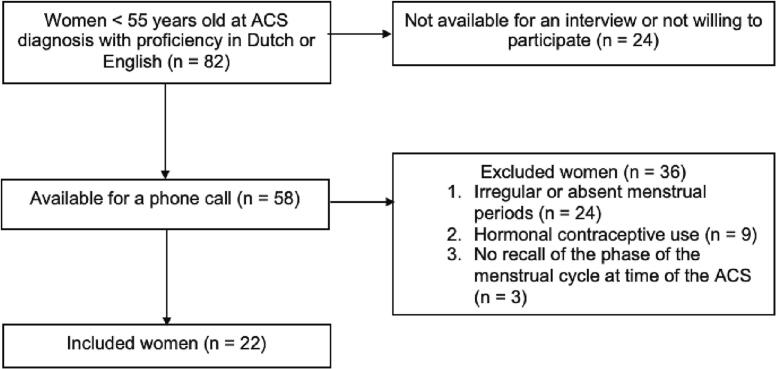
Flowchart of the inclusion and exclusion of participants.

Of the 82 women who met the inclusion criteria, 58 participated in the interview. Of these 58 women, 24 did not have a regular menstrual cycle, nine were using hormone-based contraception and three did not remember information about their menstruation at the time of ACS. The remaining 22 women were included in this case series (Fig. 1). An overview of the characteristics of all 22 women is given in Appendix B.

The 22 women had a mean age of 45.9 ± 3.4 years. All had at least one known risk factor for CVD. The most common cardiovascular risk factor was a family history of CVD (55%). The median time between the cardiovascular event and the telephone interview was 5.0 years (1.6–5.9) (see Table 1).

**Table 1:** Demographic characteristics of 22 women with Acute Coronary Syndrome.

	Women menstruating at time of event (*n* = 5)	At other time in the cycle (*n* = 17)
Characteristic	Number (%), Mean ± SD or Median (IQR)	
Age (years)	44.5 ± 3.6	46.3 ± 3.3
Time between cardiovascular event and questionnaire (years)	5.6 (1.2–6.3)	4.9 (1.7–5.8)
A history of CVD	1 (20)	1 (5.9)
Diabetes	0 (0)	3 (17.6)
Smoking	4 (80)	6 (35.3)
Obesity (BMI > 30 kg/m^2^)	2 (40)	7 (41.2)
Hypertension	2 (40)	5 (29.4)
A family history of CVD	2 (40)	10 (58.5)

Of all included women, almost a quarter (*n* = 5) were menstruating at the time of the cardiovascular event (22.7%). It was also observed that a larger proportion of women who experienced ACS while menstruating compared to other times in the menstrual cycle were smokers (80% compared with 35.3%) (Table 1).

## 4 Discussion

Almost a quarter of the study sample were menstruating at the time of their cardiovascular event (22.7%). That is higher than the percentage expected for an event unrelated to the menstrual cycle (14%), based on a regular cycle of 28 days with menstruation defined as days 1–4 of the cycle [11]. Importantly, we observed that more women who experienced ACS when their estrogen level would have been at its lowest were smokers (80% compared to 35.3%).

These findings point in the same direction as two former studies [12,13], which found a higher risk in the early follicular phase (days 1–7 of the menstrual cycle) and within the first 6 days after the onset of the menstruation. This research focused on the first four days of the menstrual cycle, when the estrogen levels are lowest. Female sex hormones (in particular estrogen) may have a variety of effects on the cardiovascular system, via many different mechanisms, such as endothelium-dependent vasodilatation [14] and increased elasticity of aortic smooth muscle cells [15]. This observation may provide more guidance for the hypothesis on the vasodilating role of estrogen, and the vasoconstrictive effects when the levels of estrogen are lowest during menstruation. The observation that there were more smokers in the group who experienced ACS when menstruating is interesting, as it is hypothesized that smoking may reduce or completely cancel the efficacy of estrogen [16].

The results of this case series may point in the direction that, as the levels of estrogen are lowest during menstruation, women may be at a greater risk of ACS at these times. Therefore, it may be important to routinely enquire about the menstrual cycle for women admitted to hospital with ACS. Only a minority of the women could recall the exact day of their menstrual cycle in retrospect. Nevertheless, it would be interesting to see whether there is a difference in the incidence of ACS on specific day(s) of the menstrual cycle in light of the hypothesis of the vasoprotective role of estrogen. The influence of the menstrual cycle could be easily examined prospectively with just a few questions in a larger cohort of women, and this would help to unravel the role of hormones in the occurrence of ACS in premenopausal women. Studies on the effect of female hormones on ACS may play an important role in assessing young women who are at greater risk for CVD, because of lower estrogen levels, for example women with Turner syndrome or premature ovarian failure, for whom hormonal replacement therapy is currently recommended by the guidelines [17,18]. The cornerstone of cardiovascular prevention consists of lifestyle advice, including smoking cessation, eating healthily and regular physical exercise, to achieve a healthy weight, as well as optimal medical therapy. It would be interesting to study whether the increased risk of venous thromboembolism associated with continuous estrogen therapy (e.g. using oral contraception without an interruption) outweighs the benefit of a possible reduction in ACS risk.

### 4.1 Limitations

First, as retrospective data were used, recall bias is a limitation of the study, as some women might not recall their menstruation years after the event. However, remarkably, only 3 of the 58 women did not remember. In addition, many women could not be reached for a telephone call because of a change in their phone number or a loss of interest in further follow-up. Finally, the sample is relatively small, as premenopausal women are a minority within the ACS population. Therefore, the results can be seen as exploratory and offering support for earlier research on this subject, and it is not possible to drawn definitive conclusions.

Despite earlier research pointing in the same way as this case series, little research has been performed on this subject whilst the burden of CVD is relatively high in this group [5]. Prospective research in a larger group of women is warranted to deepen the understanding of how the menstrual cycle influences the onset of ACS, and to determine potential additional risk factors and therapies.

## 5 Conclusion

The percentage of women who were menstruating whilst having their cardiovascular event found in this case series seems to be higher than the percentage expected for the event if it had been unrelated to the menstrual cycle. When the levels of estrogen are lowest, women may be at greater risk of ACS. Therefore, it may be important to routinely enquire about the menstrual cycle for premenopausal women admitted to hospital with an ACS. It is important to define young women who are at greater risk for ACS, as the effect on the quality of life is high. This may even lead to an assessment of the benefits of potential therapies for these women to lower their risk of ACS.

## Figures and Tables

**Table B.1:** Clinical characteristics of cases.

Case no.	Menstruation at time of event (yes/no)	Age at time of event (years)	History of CVD (yes/no)	Diabetes (yes/no)	Smoking (yes/no)	Obesity⁎) (yes/no)	Hypertension (yes/no)	A family history of CVD (yes/no)
1	No	38	No	No	No	Yes	No	Yes
2	No	44	No	Yes	No	No	Yes	No
3	No	44	No	Yes	Yes	Yes	No	Yes
4	Yes	45	No	No	Yes	Yes	No	No
5	Yes	44	No	No	Yes	No	Yes	No
6	No	46	No	No	No	No	No	Yes
7	No	46	No	No	Yes	No	No	No
8	No	49	No	No	No	Yes	Yes	No
9	No	48	No	No	Yes	Yes	No	No
10	No	48	No	No	No	No	No	Yes
11	No	50	No	No	No	Yes	No	No
12	Yes	48	No	No	No	No	Yes	Yes
13	No	50	No	No	No	Yes	No	Yes
14	No	50	No	No	No	No	Yes	Yes
15	No	49	No	No	Yes	No	No	Yes
16	No	48	No	No	No	No	Yes	Yes
17	No	42	No	Yes	No	No	No	No
18	No	47	No	No	No	Yes	No	Yes
19	No	42	Yes	No	Yes	No	Yes	Yes
20	Yes	46	Yes	No	Yes	Yes	No	No
21	No	48	No	No	Yes	No	No	No
22	Yes	39	No	No	Yes	No	No	Yes

⁎ BMI > 30 kg/m^2^.

## Appendix A Interview questionnaire

1. At the time of the cardiovascular event, were you having a regular menstrual cycle?
  a. Yes, a regular menstrual cycle ➔ question 2
  b. No, not a regular cycle ➔ question 4
  c. No, no menstruation at all ➔ question 4
2. Did you use any contraceptives?
  a. No, nothing.
  b. A condom
  c. Oral hormonal contraception
  d. Intrauterine Device (Mirena or copper)
  e. A hormonal ring
  f. An implant
  g. A contraceptive patch
3. Were you having your period during the time of the cardiovascular event?
  a. Yes
  b. No
4. For what reason you were not having a (regular) menstrual cycle?
  a. Contraceptive use
  b. I was pregnant at the moment of the event
  c. I was in the menopause at the moment of the event
  d. I had a hysterectomy
  e. I never had a menstrual cycle in my life
